# GLOSSI: a method to assess the association of genetic loci-sets with complex diseases

**DOI:** 10.1186/1471-2105-10-102

**Published:** 2009-04-03

**Authors:** High-Seng Chai, Hugues Sicotte, Kent R Bailey, Stephen T Turner, Yan W Asmann, Jean-Pierre A Kocher

**Affiliations:** 1 Department of Health Sciences Research, Mayo Clinic College of Medicine, Rochester, New York, USA; 2 Department of Medicine, Mayo Clinic College of Medicine, Rochester, New York, USA

## Abstract

**Background:**

The developments of high-throughput genotyping technologies, which enable the simultaneous genotyping of hundreds of thousands of single nucleotide polymorphisms (SNP) have the potential to increase the benefits of genetic epidemiology studies. Although the enhanced resolution of these platforms increases the chance of interrogating functional SNPs that are themselves causative or in linkage disequilibrium with causal SNPs, commonly used single SNP-association approaches suffer from serious multiple hypothesis testing problems and provide limited insights into combinations of loci that may contribute to complex diseases. Drawing inspiration from Gene Set Enrichment Analysis developed for gene expression data, we have developed a method, named GLOSSI (Gene-loci Set Analysis), that integrates prior biological knowledge into the statistical analysis of genotyping data to test the association of a group of SNPs (loci-set) with complex disease phenotypes. The most significant loci-sets can be used to formulate hypotheses from a functional viewpoint that can be validated experimentally.

**Results:**

In a simulation study, GLOSSI showed sufficient power to detect loci-sets with less than 10% of SNPs having moderate-to-large effect sizes and intermediate minor allele frequency values. When applied to a biological dataset where no single SNP-association was found in a previous study, GLOSSI was able to identify several loci-sets that are significantly related to blood pressure response to an antihypertensive drug.

**Conclusion:**

GLOSSI is valuable for association of SNPs at multiple genetic loci with complex disease phenotypes. In contrast to methods based on the Kolmogorov-Smirnov statistic, the approach is parametric and only utilizes information from within the interrogated loci-set. It properly accounts for dependency among SNPs and allows the testing of loci-sets of any size.

## Background

The genetic component of complex disorders such as hypertension, Parkinson's disease, cancer, and diabetes is believed to result from the compound effect of multiple DNA variations in different chromosomal regions. In this context, the paradigm of searching across the genome for univariate single nucleotide polymorphism (SNP) associations may not be the most appropriate or realistic strategy. A preferred approach would consider the effects of multiple SNPs jointly. Unstructured enumeration of all possible combinations of SNPs for association is computationally demanding, if not infeasible. Variable selection needs to be performed before testing such multi-locus effects due to the discrepancy between numbers of SNPs and sample size in a typical genome-wide association study. In the current work, we focused the proposed association analyses of SNPs belonging to genes that are biologically related. The criteria for grouping SNPs can be based on biological theory, expert opinion, or localization in genes that control the same functional process or are co-regulated. Such groups of SNPs will be referred to as loci-sets. We have developed a method called GLOSSI (Gene-loci Set Analysis) to score loci-sets as a function of the significance level of the individual SNPs comprising each loci-set. In what follows, we will use the terms locus and SNP interchangeably.

The idea of directly scoring a predefined set of genetic features is not new. It has sparked considerable interest in the context of gene expression data analysis since the publication of the pioneering paper by Mootha *et al*. [1,2]. These authors designated and implemented the Gene Set Enrichment Analysis (GSEA) approach to identify functionally related genes that display overall coordinated expression changes with respect to biological states or disease phenotypes. The annotated biological function is expected to be more relevant if the set is 'enriched' with genes showing good-to-moderate association signals as compared to the remaining genes.

Recently, Wang *et al*. [3] built on work of Subramanian *et al*. [2] and extended it to genotyping data. Since many SNPs can be assigned to the same gene, the authors used the best signal (biggest *χ*^2^-value) from each gene in their calculation. Similar to GSEA, enrichment of association signals was measured by using a modified Kolmogorov-Smirnov (KS) statistic and statistical significance determined through permutation testing. One drawback of the KS statistic is that it depends, in part, on the signals outside of the tested loci-set. Put another way, it assumes the 'real' causal SNPs are fully contained in a single relevant loci-set, if such a set exists. In practice, causal SNPs can probably span across multiple loci-sets, without accounting for the imperfect SNP classification that might arise, for instance, from the empirical definition of the boundary of a loci-set. Under these conditions, application of the KS statistic will result in the attenuation of the overall significance of the relevant loci-set. Another limitation pointed out by the authors is the need to carry out the computationally demanding permutation of sample labels, instead of the faster gene label permutation to properly assess the statistical significance of the KS statistic. When many loci-sets have to be tested, the computational challenge is increased since a larger number of permutations have to be performed so as to detect significant association with correction for multiple hypothesis testing.

The method we describe below addresses these two issues. GLOSSI scores loci-sets by an alternative strategy that only focuses on information from within a loci-set and allows the determination of significance level with relative computational ease.

## Results and discussion

### Fisher's combined probability test

Suppose that the data collected are from *I *independent subjects on *J *loci, where the number of loci genotyped is typically much larger than the sample size, *i.e*. *J *>> *I*. Let *y*_*i *_represents the phenotypic measurement for the *i*-th subject, *i *= 1,..., *I*, with the phenotype being understood in a broad sense as for instance a binary or multiclass label, a continuous quantity, a censored variable, or even count data. For illustration purposes, we assume here that the phenotype is binary (coded as 0/1). Using a standard encoding of the genotype as a count of the major allele, we denote the genotyping data as:



with *i *= 1,..., *I *and *j *= 1,..., *J*. Also, let *g*_*jk *_be an indicator variable indexing the *k*-th loci-set, i.e. for *j *= 1,..., *J *and *k *= 1,..., *K *(assuming there are *K *loci-sets of interest)



A measure of statistical significance is first calculated between each of the *J *loci with a chosen binary phenotype (eg case versus control). Either allele or genotype frequencies can be used as the basis for testing a locus in terms of its ability to distinguish the two phenotypic classes under study. Various statistical approaches are appropriate for deriving the p-value, from contingency-table-based methods: Fisher's exact test, Pearson's *χ*^2 ^test, or Cochran-Armitage trend test; to regression-based techniques: logistic analysis, probit analysis, or complementary-log-log analysis. These approaches could have widely different methodological assumptions, specifically on the way in which the phenotype depends on the loci (eg additive, recessive, dominant, or unconstrained). Because it is a common belief that the additive assumption is generally adequate for complex disorders, we opt for the Cochran-Armitage trend test in view of its statistical power. The trend test statistic is formulated in the Method Section. Henceforth, we denote p-value for the *j-*th locus by *p*_*j*_.

The null hypothesis of no association between *y*_*i *_and *s*_*ij *_implies that *p*_*j *_is distributed as a standard uniform random variable, taking values in the interval (0,1]. Furthermore, *t*_*j *_= -2log *p*_*j *_has a chi-square distribution with two degrees of freedom.

When SNPs are independent, the overall significance of a loci-set can be represented by a single statistic and tested on the basis of Fisher's method [4]:



where . That is, the sum of independent -2log *p*_*j *_of the *k*-th loci-set follows a chi-square distribution with degrees of freedom equal 2 times the total number of SNPs in the set. The simplicity of this approach is very appealing. In addition, it was shown by Little and Folks [5,6] that Fisher's combined probability test is asymptotically (as the number of tests is increased) Bahadur optimal. However, the assumption of independence is not tenable in a high-throughput GWAS even if only tag SNPs are used in the study. Basing inference on the independence assumption could therefore greatly inflate the apparent statistical significance of a loci-set, leading to more false positive findings. For this reason, we suggest correcting for correlation using Brown's approximation [7]:



with  in which Ω is the covariance matrix of *t*. Brown showed in his paper that the approximation works well in general except when *t*_*j*_'s are highly negatively correlated. Since correlations between *t*_*j*_s from two-sided tests can only be positive, the approximation should be adequate for most genetic association studies. Note that Ω is unknown and needs to be estimated. We chose to perform the estimation through shuffling the phenotype labels 100 times, though smaller number of permutations are often sufficient to attain a stable estimate for Ω. Details of the permutation scheme are deferred to the Method Section.

### Simulation study

In order to objectively assess the potential of the proposed methodology, we conducted a simulation study. A web-based tool, namely HapSample [8], was used to generate case-control samples with genetically realistic genotypes. We restricted the simulation to a subset of SNPs interrogated by the Sentrix^® ^HumanHap300 BeadChip [9], which consists mostly of tag SNPs. More explicitly, a total of 59,140 SNPs no more than 20 millions bases away from the end of each autosome were retained in the study. Of these, we filtered out 770 loci based on the following criteria: 737 are not in the HapMap phase I/II data [10]; 20 have a minor allele frequency (MAF) of zero according to the HapMap project (Utah population); and 13 lie near the edge of some chromosomes which possess linkage patterns (inferred from HapMap data) that are not compatible with the simulator recombination algorithm.

The genotypes of the remaining 58,370 loci were simulated with 22 settings, i.e. the null hypothesis (Scenario 0) and 21 distinct alternative hypotheses, reflecting varying numbers of SNPs associated with the case-control status and different effect sizes (see Table 1). An artificial loci-set was affixed to each scenario, but only two distinct sets were introduced to enhance comparability. We created the loci-sets and fixed causal SNPs as follows. Given a certain MAF value (5% or 25%), two SNPs at least 1 million bases apart were randomly picked from the chromosomes. We assigned a fraction of them, or one of their 'untyped' linkage disequilibrium (LD) counterparts (R^2 ^> 0.8 via Hapmap) in Scenarios 19–21, as containing a high risk allele. Note that either none or only one SNP per autosome was assumed causative in compliance with the constraint of HapSample. Moreover, we assumed additivity on the phenotype-genotype relationships and considered the causal SNPs as independent from each other. All SNPs located within 15 kilobases upstream or downstream (average size of a real gene) of the 44 selected loci constitute a loci-set. This resulted in 231 members in the MAF = 0.05 loci-set while the size of the other set was 254.

**Table 1:** Parameter specification in the simulated examples

Scenario	Number of causal SNPs	RR (OR)*	MAF^† ^of causal SNPs	Whether causal SNPs were 'genotyped'
1–3	1, 5 or 20	1.07 (1.10)	0.25	Yes
4–6	1, 5 or 20	1.34 (1.52)	0.05	Yes
7–9	1, 5 or 20	1.34 (1.50)	0.25	Yes
10–12	1, 5, or 20	1.61 (2.00)	0.05	Yes
13–15	1, 5, or 20	1.61 (1.94)	0.25	Yes
16–18	1 or 5	2.00 (2.67)	0.25	Yes
19–21	1, 5, or 17^‡^	1.61 (~1.94)	~0.25	No

*RR (OR) = relative risks (odds ratio) when a loci carries two disease alleles, assuming an additive model; ^†^MAF = minor allele frequency of the Utah samples in the HapMap project. ^‡^Three out of the 20 original causal SNPs, generated under the case where MAF equals 0.25, are not in high LD with any SNP genotyped in the HapMap phase I/II project. Disease prevalence and crossover rate are fixed at 25% and 1.0 centiMorgan, respectively, in all simulations.

**Figure 1:**
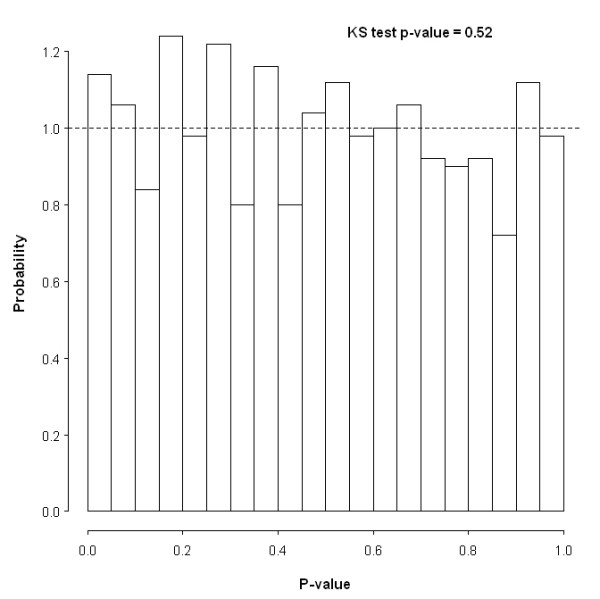
**Histogram of p-values acquired under the null hypothesis (Scenario 0) based on 1000 simulated data sets of 200 cases and 200 controls**. The dashed line is the expected theoretical height of a bar if no SNP in the loci-set was related to the case-control labels.

Every single simulation setup was replicated a thousand times for each of these case-control sizes: 200–200, 400–400 and 1000–1000. In other words, a total of 66 thousand independent data sets were generated. GLOSSI was run on the simulated data sets one-at-a-time and the resulting significance levels of the hypothetical loci-sets were stored so as to evaluate statistical power and type I error rate. These were derived as the proportion of loci-set p-values achieving a smaller numerical value than 0.05. As would be anticipated, the distribution of p-values under the null hypothesis closely resembles the standard uniform distribution (Figure 1). The empirical type I error rates, calculated under 0.05 as well as two other popular nominal levels, are presented in Table 2. All type I error rates are within the 95% confidence intervals of the specified levels (*α *= 0.05, 95% CI 0.036 – 0.064; *α *= 0.001, 95% CI 0.004 – 0.016; *α *= 0.001 95% CI 0 – 0.003). This leads us to conclude that GLOSSI offer adequate results in the true null scenario.

**Table 2:** Estimated type I error rates for GLOSSI in the simulated examples

Total sample size	Nominal rate, *α*	Proportion of p-value <*α*
400	0.05	0.057
	0.01	0.011
	0.001	0

1200	0.05	0.045
	0.01	0.012
	0.001	0.002

2000	0.05	0.049
	0.01	0.010
	0.001	0.003

**Figure 2:**
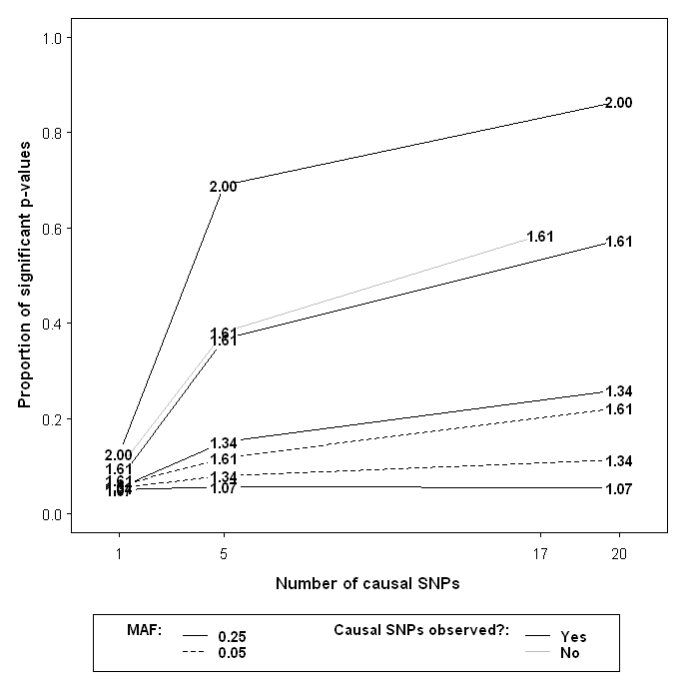
**Statistical power estimated using 200 cases and 200 controls across a range of experimental settings**. x-y coordinates of the numbers within the plot represent number of causal SNPs and power respectively for individual simulated examples. Relative risks (RR) are denoted by the numbers themselves. Cases with the same RR value and MAF of 0.25 are linked using solid lines while those having MAF of 0.05 are joined by dashed lines. The lines are colored grey if causal SNPs were not genotyped; black otherwise.

**Figure 3:**
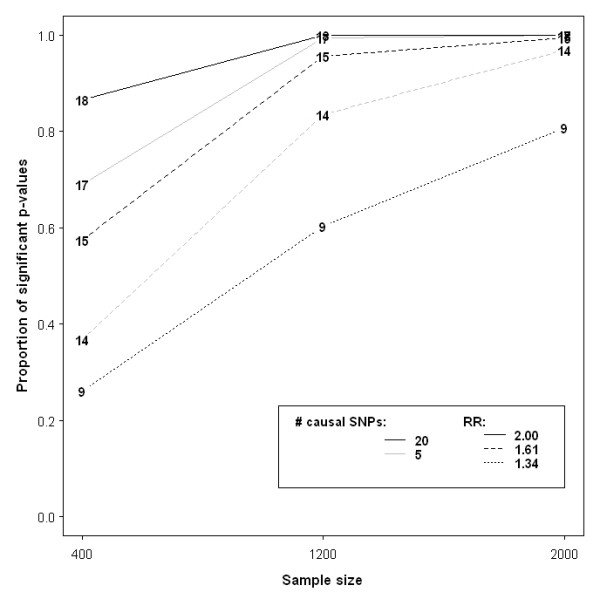
**Plot of power versus sample size**. Only scenarios surpassing 80% power in the case of 2000 samples are illustrated, except for Scenarios 20 and 21 where their curves closely resemble those from Scenarios 14 and 15. Integers within the plot denote the scenario number (see Table 1).

The results for 200 affected and 200 unaffected samples under the varying alternative hypotheses are graphically documented in Figure 2. Despite having a smaller fraction of causal SNPs than the corresponding MAF = 0.05 set, the enriched MAF = 0.25 loci-set is more readily identified by GLOSSI as significant. It is surprising to see that relatively higher power is obtained in cases where causal SNPs are not 'genotyped'. This is counterintuitive and likely to occur by chance, but it requires further investigation. It should also be noted that the method displays very low statistical power when only 1 causal SNP was included in the loci-set. The capability of detecting one-SNP enrichment stays below the standard 80% cut-off level for the other sample sizes considered in this study (data not shown). Figure 3 shows the relationship between sample size and power for some representative scenarios in which sufficient power was attained at the total sample size of 2000. Given that we fixed less than 10% of the SNPs in the MAF = 0.25 loci-set as causative, GLOSSI exceeds 80% power when i.) relative risk (RR) is 2 and sample size is 400, ii.) RR = 1.61 with 1200 samples, or iii.) RR = 1.34 with 1000 cases and 1000 controls. In summary, higher RR, larger MAF, increased number of causal loci and bigger sample size all have a positive impact on the power.

It is of interest to compare the performance of GLOSSI against the modified KS approach proposed by Wang *et al*. [3]. To this end, eight additional non-overlapping regions (hypothetical genes) were randomly picked from each chromosome. We altered the extension of the newly selected regions to either double, equal or halve the size of the hypothetical genes in the loci-set. These were used to create a reference distribution in the modified KS test. Without loss of generality, we focused the comparison on the null and two alternative hypotheses with the use of our data set of 200 cases and 200 controls. Scenarios 17 and 18 were chosen here because their powers were near 80% in the case of GLOSSI.

Outputs from the modified KS test on the basis of both phenotype and gene label permutations under the null hypothesis are summarized in Table 3. When there is inequality in the gene sizes in and out of the loci-set, gene label shuffling, though less computationally burdensome, can lead to substantial deviation from the nominal type I error rate of 5%. It offered satisfactory result when the size of all the hypothetical genes was set to be the same. In contrast, resampling case-control status appears adequate for the null distribution regardless of the relative size of genes. However, size of genes did have an influence on the power of the modified KS statistic in the two tested non-null hypotheses even when sample label permutation was applied. Proportion of significant p-values (relative size of genes: out/in) was calculated to be 48.6% (1/2), 59.8% (1/1) and 70.8% (2/1) for Scenario 17 and 84.7% (1/2), 85.5% (1/1) and 85.8% (2/1) for Scenario 18. The KS statistic seemed to parallel GLOSSI (power = 69.0% – Scenario 17; and 86.6% – Scenario 18) in power when larger genes were used as reference.

**Table 3:** Estimated type I error rates for the modified KS statistic

Type of permutation	Relative size of genes: out/in loci-set	Proportion of p-value < 0.05	95% CI
Phenotype	50%	0.052	(0.038,0.066)
	100%	0.053	(0.039,0.067)
	200%	0.051	(0.037,0.065)

Gene	50%	0.27	(0.24,0.29)
	100%	0.046	(0.033,0.059)
	200%	0.004	(0,0.008)

We speculated in the Background Section that causal loci in genes not belonging to the query loci-set can dilute the degree of significance and, therefore, statistical power of the KS statistic. Moreover, the power should drop as the fraction of genes with causal SNPs outside of the loci-set increases. To test these, we reassigned two SNPs (with MAF = 0.25) in different genes of the reference set as causative. Since HapSample can only handle 1 causal locus per chromosome, we study the change in the proportion of loci being causative by decreasing the number of hypothetical genes not in the loci-set from 176 to 88 and then to 44. Results from case-control label shuffling are presented in Table 4. As expected, Scenario 18 with more SNPs having high risk alleles is less susceptible to the 'contaminants'. Although the simulation setup is somewhat artificial, it supports our claims in general.

**Table 4:** Power of the modified KS statistic when two genes in the reference set consist of a causal SNP

Number of genes outside of loci-set	Relative size of genes: out/in loci-set	Proportion of p-value < 0.05	
		
		Scenario 17	Scenario 18
	50%	0.464	0.842
176	100%	0.581	0.854
	200%	0.684	0.860

	50%	0.371	0.804
88	100%	0.495	0.829
	200%	0.629	0.854

	50%	0.145	0.683
44	100%	0.187	0.732
	200%	0.161	0.773

### Antihypertensive response example

GLOSSI was used in this example to identify potentially instructive loci-sets for their influences on diastolic blood pressure (DBP) response to hydrochlorothiazide in the Genetic Epidemiology of Responses to Antihypertensives study (GERA) [11]. On the basis of the age and baseline DBP-adjusted distribution of DBP response, the study group was partitioned into race-and-gender specific "good", "intermediate" and "poor" responders. Raw intensity data obtained via the Affymetrix GeneChip^® ^Human Mapping 100 K Set [12] were available for 194 African Americans (97 good and 97 poor responders, 50 women and 47 men in each of these response groups) and 195 non-Hispanic Caucasians (98 good – 42 females and 56 males; and 97 poor – 42 females and 55 males responders). Genotype calls were made through the use of Dynamic Modeling algorithm [13]. The following SNPs were excluded from the analysis: on the X-chromosome, monomorphic, MAF < 2%, "call"-rate < 80% or deviated from Hardy-Weinberg equilibrium at p < .001. Thus our illustration is based on 102,334 and 95,221 post-filtering SNPs in the black and white samples, respectively. See Turner *et al*. [14] for a detailed description of the study design and procedures taken to preprocess the Affymetrix data. We derived loci-sets from 1412 generic and human-specific functional sets of the publicly accessible Molecular Signature Database (MSigDB version 2.1 [15]). Only SNPs located within 5000 base pairs upstream or downstream of a gene (defined using Affymetrix build na24 annotation files) were considered relevant to that gene. Table 5 reports the most significant loci-sets from applying GLOSSI on all non-empty loci-sets (1405 for Whites and 1404 for Blacks). q-values [16,17] were calculated to guard against the cost of multiple hypothesis testing. This provides an expected proportion of false positives among loci-sets with unadjusted p-values at least as extreme as the current set of interest.

**Table 5:** Loci-sets with unadjusted p-value no greater than 0.1% in the antihypertensive response example

Loci-set	MsigDB ID	No. SNP	No. relevant gene	p-value	q-value
*Non-Hispanic white*					
TPO signaling pathway	c2:338	48	10	0.0001	0.035
Erk1/Erk2 Mapk signaling pathway	c2:178	74	16	0.0001	0.035
Sprouty regulation of tyrosine kinase signals	c2:316	36	10	0.0001	0.035
Multiple antiapoptotic pathways from IGF-1R signaling lead to bad phosphorylation	c2:214	24	8	0.0002	0.035
PTEN pathway	c2:557	30	8	0.0002	0.035
Transcription factor CREB and its extracellular signals	c2:152	83	16	0.0002	0.035
Growth hormone signaling pathway	c2:198	50	11	0.0002	0.035
PTEN dependent cell cycle arrest and apoptosis	c2:292	24	8	0.0003	0.035
Upregulated in acute rejection transplanted kidney biopsies	c2:834	132	25	0.0003	0.035
IL 3 signaling pathway	c2:223	18	6	0.0003	0.035
Trka receptor signaling pathway	c2:339	41	5	0.0003	0.035
IL-2 receptor beta chain in T cell activation	c2:222	40	11	0.0004	0.035
B cell antigen receptor	c2:569	49	18	0.0004	0.035
IL 4 receptor signaling in B lymphocytes	c2:563	39	12	0.0004	0.035
Calcium signaling by HBx of Hepatitis B virus	c2:569	16	4	0.0005	0.035
Glycogen processing	c2:602	39	8	0.0005	0.035
IGF-1 signaling pathway	c2:213	31	9	0.0005	0.035
Down regulated following Apc loss	c2:1048	156	32	0.0005	0.035
Liver selective	c2:979	300	104	0.0005	0.035
TrkA receptor	c2:559	19	6	0.0006	0.035
Inhibition of cellular proliferation by gleevec	c2:199	37	10	0.0006	0.035
IL 6 signaling pathway	c2:226	25	8	0.0006	0.035
Insulin signaling pathway	c2:229	26	8	0.0007	0.039
Upregulated in fibroblasts following infection with human cytomegalovirus	c2:1269	131	24	0.0008	0.040
Down regulated by both curcumin and sulindac in SW260 colon carcinoma cells	c2:1412	50	10	0.0010	0.047
Upregulated by TPA in resistant HL-525 cells	c2:1679	90	19	0.0010	0.048
					
*African American*					
Upregulated by UV-B light in epidermal keratinocytes	c2:1717	55	12	0.0004	0.56
Upregulated in well functioning transplanted kidney biopsies	c2:836	1347	285	0.0009	0.63

GLOSSI reported 26 loci-set with a q-value lower than 5% in Whites but no loci-set passed this cutoff in Blacks (Table 5). The size of the 26 loci-sets ranges from 16 to 300 SNPs. This result is quite encouraging since single SNP methods previously applied to the same datasets could not detect any SNP that was statistically significantly associated with DBP response to hydrochlorothiazide (unpublished results). Among the top ranking loci-sets of the populations, two were derived from the same gene expression experiment of kidney transplant biopsies [18]. These loci-sets are 'upregulated in acute rejection transplanted kidney biopsies' (MsigDB ID = c2:834, p-value = 0.0003) for non-Hispanic Caucasians and 'upregulated in well functioning transplanted kidney biopsies' (MsigDB ID = c2:836, p-value = 0.0009) for African Americans. Although biological interpretation of the results is not straightforward, one can hypothesize that genes in those two loci-sets are related to kidney pathophysiology or normal physiology and, therefore, may be relevant to sodium excretion, blood pressure regulation, and DBP response to diuretic therapy. One could also speculate that the different physiological mechanisms indexed by these two loci-sets are consistent with known differences in diuretic response between Black and White individuals with hypertension.

Other loci-sets are less informative and harder to interpret. Inspection of their names suggests that several of the significant loci-sets in Whites could conceivably be involved in regulation of antihypertensive drug response. These include 'growth hormone signaling pathway' (MsigDB ID = c2:198), 'calcium signaling by HBx of Hepatitis B virus' (MsigDB ID = c2:569), and 'insulin signaling pathway' (MsigDB ID = c2:229). The relationship of some other loci-sets with DBP response to hydrochlorothiazide requires a more speculative interpretation. For example, a few of them appear to be related to cell growth regulation but with no obvious relationship to blood pressure. However, a possible connection could exist through mitogenic hormones that are often vasoconstrictive and antinatriuretic and, therefore, would elevate blood pressure (eg, angiotensin II). Conversely, vasodilating and natriuretic hormones that lower blood pressure are often anti-mitogenic (eg, atrial natriuretic peptide).

## Conclusion

The GLOSSI methodology for scoring loci-sets (a priori defined groups of SNPs) overcomes limitations of commonly-used single SNP approaches. The origin of a loci-set facilitates the interpretation of statistical outputs, providing a biological understanding of the mechanisms that underlie diseases or other phenotypes of interest. In contrast to the approach of Wang *et al*. [3], the proposed procedure is parametric: it assumes that p-values from individual SNPs follow a standard uniform distribution under the null hypothesis of no association and infers statistical relevance of each loci-set against a *χ*^2 ^distribution. Consequently it has the advantage of computational speed, demands measurements only of SNPs within the query loci-set, and imposes no constraint on the size of the set. Although we only focus on binary phenotypes in this communication, the technique is general and equally applicable to other kinds of outcomes or any types of genome-scale data. In particular, the locus-specific p-values could be generated by statistical methods equipped with the ability to control for the presence of covariates (eg age, gender, etc). Appropriate adjustment for additional covariates would allow more accurate estimation of the true genotype-phenotype effect. The performance of the proposed method was evaluated by using computer simulated data as well as data from an antihypertensive pharmacogenomic study. In the simulation study, GLOSSI yielded the anticipated type-I error rate when no SNP in the loci-set was related to the binary outcome. Also, it demonstrated sufficiently high power for detecting loci-sets in which a fair number of SNPs (< 10%) had moderate to large effect sizes and intermediate MAF values. In the real data example, the proposed method appears to have been able to identify novel loci-sets not previously known or suspected to be involved in blood pressure regulation or antihypertensive drug response.

The lack of firm biological interpretation in the antihypertensive response example underlines one of the limitations of our method. Although GLOSSI is capable of detecting relevant loci-sets as demonstrated in the simulation experiment, its usefulness depends directly on the definition and availability of loci-sets when applying it to biological data. The currently available functionally annotated loci-sets are biased toward groups of genes involved in cancers since most of them were derived from such disease studies but very few of them focus on blood pressure or kidney-related investigations. Undoubtedly, more annotated and curated loci-sets will be available over time, which in turn will increase the applicability of GLOSSI for a given disease phenotype. The definition of a loci-set itself can also be challenged. The current assignment of SNPs to a gene, according to fixed physical distance boundaries from that gene, might not be optimal, not even in principle, let alone given the uncertainty in determining the appropriate fixed distance.

It must be stressed that GLOSSI only accounts for the additive, independent effect of individual SNPs and, therefore, ignores possible biological interactions that might exist. The joint effect of SNPs within a loci-set can be captured using multivariate methods [19-21]. However, a fair comparison of multivariate models derived from various loci-sets is hard to achieve since it demands sample label permutation testing. More specifically, the statistical model needs to be rebuilt for every loci-set in each permutation, which quickly becomes impractical as the numbers of loci-sets and permutations increase. Other complications that might arise during the application of multivariate analysis include overfitting and model instability. To balance the need for joint effects modeling with computational time effectiveness, one can envision developing a hierarchical approach that first uses GLOSSI for rapid identification of significant loci-sets followed by more extensive multivariate modeling. This approach is currently being investigated in our group.

## Methods

### Cochran-Armitage trend test

For the *j*th locus, the trend test statistic can be written as:



where







with *δ*(.) signifies an indicator function taking value one if its argument is correct and zero otherwise. The null hypothesis of the test is no linear trend in the proportion of group memberships at each of the three SNP genotypes, i.e. the proportion of 0/1 class distinction is the same for all levels. The statistic follows a standard normal distribution under this null hypothesis. Hence the evidence of association (p-value) between *y*_*i *_and *s*_*ij *_can be inferred by comparing *T*_*j*_^2 ^to a *χ*^2 ^distribution with 1 degree of freedom, i.e. this will be a two-sided test.

### Estimating covariance matrix by permutation

The existence of local LD implies that *t*_*j*_, *j *= 1,..., *J*, are not independent. Their covariance matrix, Ω, under the null hypothesis can be estimated through the use of permutation as follows. Assume that the phenotype measurements are independently and identically distributed. The subscript of *y*_1_, *y*_2_,..., *y*_*I *_are first shuffled reiteratively. One could generate either all *I*! permissible permutations or just a random sample of them. Then recalculate *t*_*j *_for all loci over every permuted datasets. Each of the resulting set of *t*-values represents a joint observation from the sampling distribution of *t *= (*t*_*j*_,..., *t*_*j*_)^T ^that is consistent with the null hypothesis. Given enough permutations, the empirical covariance of the *t*-values from the above should approximate Ω. Note that because not all SNPs are assigned to loci-sets, it is more computationally efficient to perform the calculations only on the relevant loci.

## Authors' contributions

HSC drafted the manuscript, led the development of the statistical method and performed the analysis of the simulated and experimental data. HS participated in the development of the statistical method and in the design and building of the simulated data sets. KRB advised and supervised the development of the statistical method. STT provided the experimental data, disease domain expertise, and support for the biological interpretation. YWA provided access to loci-sets and bioinformatics expertise. JPAK helped to draft the manuscript and authorized publication.
